# Elevated total cholesterol in healthy adults mediates the association between impaired thyroid hormone sensitivity and hyperuricemia

**DOI:** 10.3389/fendo.2025.1625843

**Published:** 2025-08-28

**Authors:** Xiying Huang, Daojun Yu

**Affiliations:** ^1^ Graduate School, Zhejiang Chinese Medical University, Hangzhou, China; ^2^ Department of Laboratory Medicine, The Second Affiliated Hospital of Zhejiang University School of Medicine,Hangzhou, China

**Keywords:** impaired thyroid hormone sensitivity, hyperuricemia, healthy adults, total cholesterol, mediation

## Abstract

**Purpose:**

This study aims to explore the association between impaired thyroid hormone sensitivity and hyperuricemia, and to analyze the potential mediating role of total cholesterol (TC), thereby providing a new theoretical basis for the early prevention and intervention of hyperuricemia.

**Methods:**

This study utilized health check-up data from Health Examination Management Center of the second affiliated hospital of Zhejiang University School of Medicine, collected between 2019 and 2020. The dataset included 80568 participants with normal thyroid function, no family history of thyroid disorder, and no history of thyroid hormone or lipid-lowering medication use. The extent of impaired thyroid hormone sensitivity was assessed using thyroid hormone sensitivity indices, such as the Thyroid Feedback Quantile Index (TFQI), Parameter Thyroid Feedback Quantile Index (PTFQI), Thyroid Resistance Index (TT4RI), and Thyroid Stimulating Hormone Index (TSHI). Logistic regression models were employed to analyze the relationship between thyroid hormone sensitivity indices and hyperuricemia, while mediation analysis was conducted to quantify the mediating effect of total cholesterol.

**Result:**

Among the 22843 participants, 6597(28.88%) were diagnosed with hyperuricemia. The thyroid hormone sensitivity indices (TFQI, PTFQI, TT4RI, and TSHI) were notably elevated in the hyperuricemia group compared to the non-hyperuricemia group (P<0.001). A dose-response relationship was observed between thyroid hormone sensitivity indices and hyperuricemia, and this association remained noticeable after adjusting for factors such as age, gender, BMI, hypertension, and diabetes. Mediation analysis indicated that total cholesterol significantly mediated the association between impaired thyroid hormone sensitivity and hyperuricemia, with a mediation proportion ranging from6.89% to 18.42%.

**Conclusion:**

This study is the first to uncover a new relevance by which impaired thyroid hormone sensitivity mediates hyperuricemia through total cholesterol. This finding provides fresh epidemiological evidence for the interplay between thyroid dysfunction and hyperuricemia and suggests that monitoring thyroid hormone sensitivity indices in high-risk populations for hyperuricemia might be conducive in early risk stratification. Moreover, lipid-lowering treatment in subclinical thyroid dysfunction patients may simultaneously enhance uric acid metabolism, offering creative insights for the precise prevention and management of hyperuricemia.

## Introduction

Hyperuricemia refers to the phenomenon where fasting serum uric acid levels exceed the upper reference range on two separate and non-consecutive occasions under normal purine dietary conditions. Uric acid is the final metabolic product of purine compounds in humans, and uric acid metabolism maintains a dynamic equilibrium in normal circumstances. However, abnormal purine metabolism or reduced uric acid excretion can lead to hyperuricemia. In the general population of China, approximately 13.3% of individuals suffer from hyperuricemia that amounts to around 177 million people; whereas some island areas report rates above 20%, and in certain regions, the prevalence among males can exceed 30% (1). The prevalence of hyperuricemia is notably more prevalent in males than females, with monitoring data from 2018-2019 showing a male prevalence of 24.5%, and 32.3% among young men aged 18-29 years (2). Hyperuricemia serves as a crucial biochemical basis for gout and is also liked to metabolic syndrome, cardiovascular diseases, and chronic kidney disease.

hypothyroidism is associated with dyslipidemia, diabetes, fatty liver, and hyperuricemia (3). Therefore, in our study, we introduced the thyroid hormone sensitivity index (also known as the thyroid hormone sensitivity impairment index, which describes and quantifies the sensitivity of tissues to circulating thyroid hormones) to analyze the relationship between thyroid hormone function (mainly FT3 and FT4) and hyperuricemia.

This study investigated the correlation between the degree of thyroid hormone sensitivity impairment and hyperuricemia in a healthy adult population. By using mediation effect analysis with total cholesterol (TC) as a quantifier, we explored the direct and indirect associations between them. Ultimately, we propose a viewpoint that we may be able to intervene in thyroid hormone sensitivity impairment and hyperuricemia by controlling serum total cholesterol (TC). This could provide a potential intervention measure for the metabolic health of patients with thyroid hormone sensitivity impairment.

**Figure 1:**
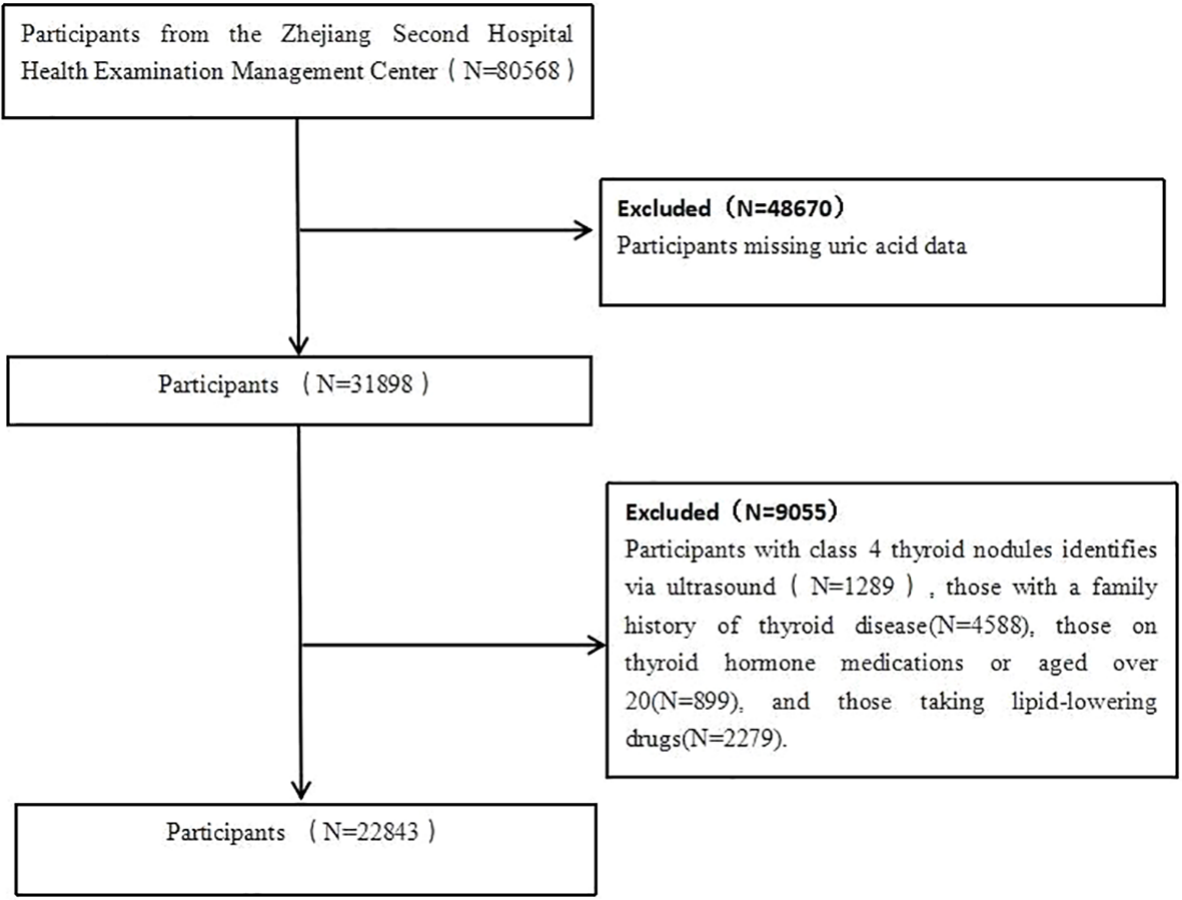
Flow of participants in current study.

Using data from the second affiliated hospital of Zhejiang University School of Medicine Health Examination Management Center, this study conducts a secondary analysis on participants who underwent health checks between 2019 and 2020, encompassing a total of 180,568 individuals. Exclusions included those missing uric acid data, individuals with class 4 thyroid nodules identifies via ultrasound, those with a family history of thyroid disease, those on thyroid hormone medications or aged over 20, and those taking lipid-lowering drugs. Ultimately, 80568 participants were included in the study (**Figure 1**). This study was approved by the Ethics Committee of the Second Affiliated Hospital, Zhejiang University School of Medicine (Approval No: 2021LSYD0360).

### Data collection and definitions

Demographic characteristics (age, gender), health-related information (medical history and medication use) were collected by our trained staff. Self-reported medical history included hypertension, diabetes, and thyroid dysfunction. Physical examinations involved measuring height, weight, and blood pressure. Body mass index (BMI) was calculated using the formula: weight (kg) divided by the square of height (m). A total of 22843 participants were included in this study. After resting for at least 10 minutes, systolic and diastolic blood pressures were measured on the right arm with a sphygmomanometer, and the average of two measurements was recorded. Fasting blood glucose and serum uric acid concentrations were measured following an overnight fast (at least 8 hours), using an automated biochemical analyzer (AU5800). Serum TSH and FT4 levels were determined by electrochemiluminescence immunoassay on an Abbott immunoassay automatic analyzer. The specific reference ranges for TSH and FT4 are 0.2–6.00 mIU/L and 10.00–25.61 pmol/L, respectively.

Hypertension was defined as a systolic blood pressure ≥140 mmHg, or diastolic blood pressure ≥90 mmHg, or self-reported hypertension diagnosis, or the use of any antihypertensive medication. In the Chinese population, hypercholesterolemia was characterized as a total cholesterol level exceeding 6.2 mmol/L. Hypertriglyceridemia was identified as a fasting plasma triglyceride level exceeding 1.7 mmol/L. Hyperuricemia was determined as serum uric acid levels greater than 360 μmol/L for females and greater than 420 μmol/L for males.

Thyroid hormone sensitivity was assessed using several indices. The thyroid feedback quantile index (TFQI) was calculated as the empirical cumulative distribution function (cdf) of FT4 minus (1-cdf TSH). Additionally, the parameter thyroid feedback quantile index (PTFQI) was computed using the standard normal cumulative distribution function Φ((FT4-μFT4)/σFT4) - (1- Φ((Ln TSH-μLn TSH)/σLn TSH)) (4). The population parameters were as follows: μFT4 = 15.869, σFT4 = 2.323, μLn TSH = 0.640, and σLn TSH = 0.482. The values for TFQI and PTFQI range from -1 to 1, with higher positive values indicating poorer thyroid hormone sensitivity. The thyroid-stimulating hormone resistance index (TT4RI) was calculated as FT4 (pmol/L) × TSH (mIU/L) (5). The thyroid-stimulating hormone index (TSHI) was derived using the formula: Ln TSH (mIU/L) + 0.1345 × FT4 (pmol/L) (6). Higher values for TT4RI and TSHI signify a greater degree of impaired thyroid hormone sensitivity.

Normally distributed variables were presented as mean (standard deviation), skewed variables as median (interquartile range), and categorical variables as frequency (proportion). Independent t-tests or Mann-Whitney U tests were employed to compare continuous variables, while chi-square tests were adopted to compare categorical variables. The distribution of thyroid hormone sensitivity indices was represented by violin plots. Logistic regression models were used to study the association between impaired thyroid hormone sensitivity and hyperuricemia. Odds ratios (OR) and 95% confidence intervals (CI) (7) were computed after adjusting for age, gender, hypertension, diabetes, and other factors. The dose-response relationship between thyroid hormone sensitivity indices and hyperuricemia, both unadjusted and adjusted, was analyzed using restricted cubic spline functions with three knots positioned at the 10th, 50th, and 90th percentiles. Thyroid hormone sensitivity analysis was performed on 22843 participants with serum total cholesterol data.The sample size is far larger than what is appropriate for this study. In brief, the thyroid hormone sensitivity index serves as the predictor variable (X), serum total cholesterol as the mediator variable (M), and hyperuricemia as the outcome variable (Y). The analysis included four steps: (1) establish the correlation between X and Y (model Y = βTot X) (βTot = total effect); (2) establish the association between X and M (model M = β1 X) (β1 = indirect effect 1); (3) determine which portion of Y can be explained by controlling X (model Y = β2m + βDir X) (β2 = indirect effect, βDir = direct effect); (4) calculate the proportion of the indirect or mediated effect: mediated effect (%) = (β1 × β2/βTot) × 100%. This method has been widely used in previous studies to quantify mediation effects (8, 9).

All statistical analyses were performed utilizing R software (version 4.2.1). Mediation analysis was conducted using the “Mediation” package. A two-sided p-value < 0.05 was considered statistically significant.

## Results

### Population characteristics

Among the 22843 participants, 14148 were male (61.90%) and 8695 were female (38.10%). The mean (SD) age was 46.32 (13.34) years. A total of participants had hyperuricemia (28.88%).Hyperuricemia is significantly more prevalent among males. Participants with hyperuricemia exhibited higher BMI and FT4 levels. The probability of hypertension was notably greater in participants with hyperuricemia compared to those without (**Table 1**). Obviously, HDL was significantly lower and LDL was significantly higher in the hyperuricemia group compared to the non-hyperuricemia group. Besides, FT4 and TSH levels were markedly elevated in the hyperuricemia group, resulting in significantly higher thyroid hormone sensitivity indices (TFQI, PTFQI, TT4RI, and TSHI) when compared to the non-hyperuricemia group. As depicted in **Table 1**, the proportion of obesity, diabetes, hypercholesterolemia, and hypertriglyceridemia in the hyperuricemia group was significantly higher than the normal population. Furthermore, the BMI of the hyperuricemia group was significantly higher than that of the non-hyperuricemia group.

**Table 1:** Basic characteristics of the participants.

Item	Overall	Normouricemia	Hyperuricemia^e^	P value
Participants, No	22843	16246 (71.11)	6597 (28.88)	
Age, mean (SD), y	46.32 (13.34)	46.09 (13.19)	46.86 (13.86)	0.000
Sex				
Female	8695 (38.1)	7743 (47.7)	952 (14.4)	0.000
Male	14148 (61.9)	8503 (52.3)	(85.6)	0.000
Hypertension^a^	3139 (13.7)	1940 (11.9)	1199 (18.20)	0.000
Diabetes^b^	917 (4.00)	598 (3.7)	319 (4.8)	0.004
Hypertriglyceridemia^d^	6174 (27.03)	4123 (25.38)	2434 (36.90)	0.000
Hypercholesterolemia^c^	5738 (25.12)	3931 (24.20)	2019 (30.61)	0.000
HDL mean (SD) mmol/L	1.39 (0.31)	1.44 (0.31)	1.27 (0.28)	0.000
LDL mean (SD) mmol/L	2.58 (0.70)	2.52 (0.69)	2.74 (0.70)	0.000
Total cholesterol mean (SD), mmol/L	5.04 (0.99)	4.97 (0.98)	5.20 (1.01)	0.000
Triglycerides mean (SD),mmol/L	1.29 [0.49]	0.77 [0.41]	1.02 [0.62]	0.000
FT4, median [IQR], pmol/L	12.79 [12.01,13.6]	12.42 [11.34,13.8]	12.64 [12.08,13.7]	0.000
TSH, median [IQR], mIU/L	1.44 [1.03, 2.04]	1.44 [1.02, 2.03]	1.45 [1.04, 2.04]	0.291
BMI, mean (SD), kg/m2	23.77 (3.28)	23.4 (3.12)	25.97 (3.03)	0.000
UA, mean (SD), μmol/L	357.74 (90.68)	313.33 (59.43)	467.12 (54.12)	0.000

SD, standard deviation; IQR, interquartile range; TC, cholesterol; TSH, thyrotropin; FT4, free thyroxine; UA, uric acid; SI, conversion factors: To convert FT4 to ng/dL divided by 12.871, BMI is calculated as weight in kilograms divided by height in meters squared.

a Hypertension was defend as systolic blood pressure ≥ 140 mmHg or diastolic blood pressure ≥ 90 mmHg or self-reported diagnosis history of hypertension or use of any anti-hypertensive medication.

b Diabetes was defined as fasting glucose ≥ 7.0 mmol/L or self-reported diagnosis history of diabetes or using any glucose-lowering medication e Hyperuricemia was defined as serum uric acid ≥ 360 μmol/L in females and ≥ 420 μmol/L in males or the use of uric acid-lowering medications.

c Hypercholesterolemia is defined as total cholesterol exceeding 6.2 mmol/L in the Chinese population.

d Hypertriglyceridemia (HTG) is a type of dyslipidemia characterized primarily by elevated levels of triglycerides in the blood. It is defined as a fasting plasma triglyceride level exceeding 1.7 mmol/L.

e Hyperuricemia was defined as serum uric acid ≥ 360 μmol/L in females and ≥ 420 μmol/L in males or the use of uric acid-lowering medications.

### Thyroid hormone sensitivity impairment and hyperuricemia

**Figure 2:**
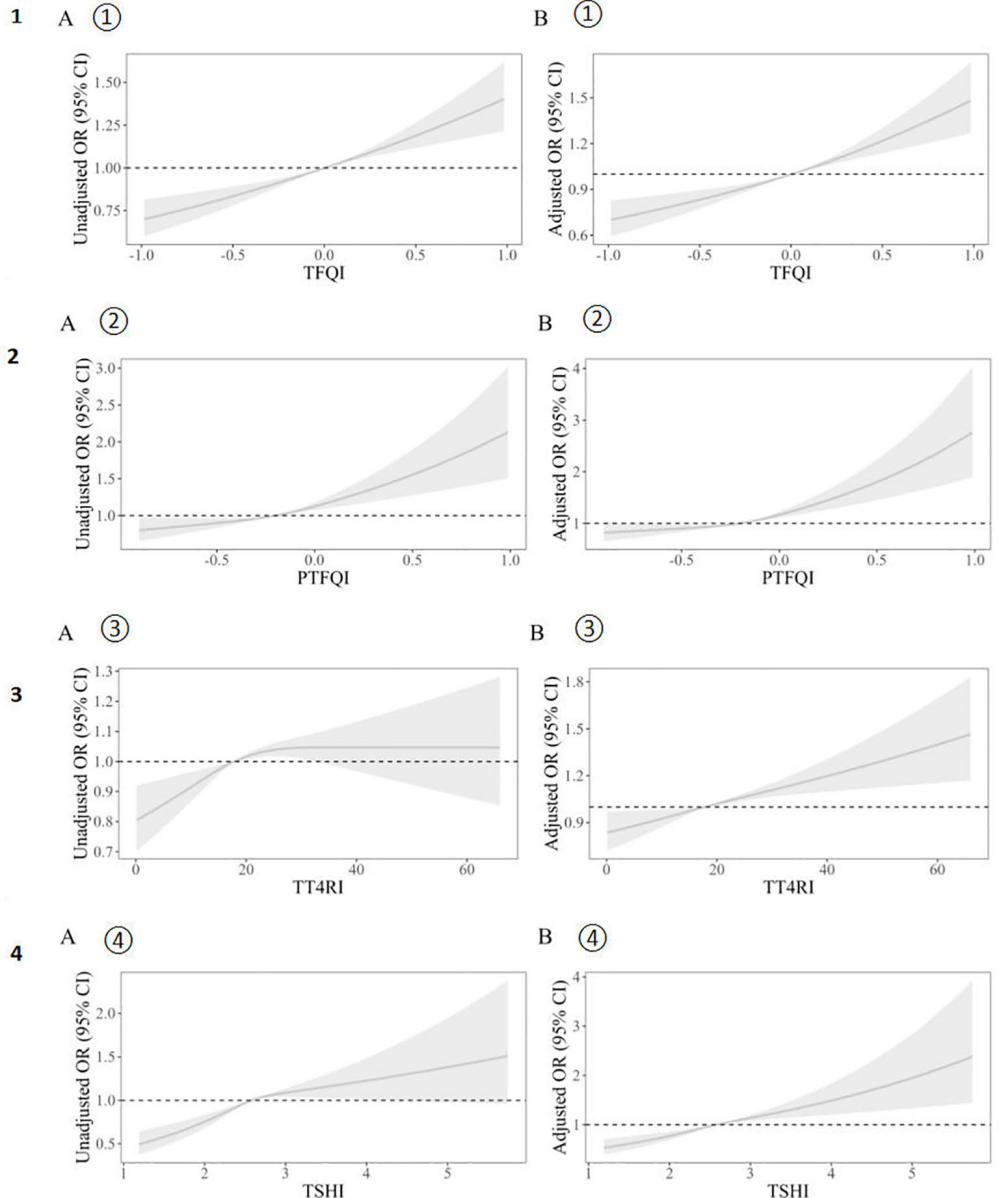
Unadjusted **(A)** and adjusted **(B)** dose–response relationship between thyroid hormones sensitivity indices and hyperuricemia. The distribution of thyroid hormone sensitivity indices was represented by violin plots. Logistic regression models were used to study the association between impaired thyroid hormone sensitivity and hyperuricemia. Unadjusted (A① A② A③ A④)and adjusted(B① B② B③ B④) dose–response relationship between thyroid hormones sensitivity indices and hyperuricemia using the restricted cubic spline method. A restricted cubic spline regression model was conducted using 3 knots at the 10th, 50th, and 90th percentiles; the results were adjusted for sex, age (continuous), hypertension (categorical), BMI and diabetes (categorical). TFQI, thyroid feedback quantile-based index; PTFQI, parametric thyroid feedback quantile-based index; TT4RI, thyrotrophic thyroxine resistance index; TSHI, thyroid-stimulating hormone index.

**Figure 2** illustrates the correlation between thyroid hormone sensitivity indices and hyperuricemia, both pre- and post-adjustment. The results were adjusted for gender, age (continuous variable), hypertension (categorical variable), BMI, and diabetes (categorical variable). As can be seen in **Figure 2**, the correlation between the thyroid hormone sensitivity index and hyperuricemia becomes much stronger after adjustment. The data distribution is also more concentrated. **Table 2** presents two models. Model 1 is the crude model, while Model 2 is adjusted for sex, age (continuous), BMI, hypertension (categorical), and diabetes (categorical). By excluding these confounding factors, the data becomes more objective. The four thyroid hormone sensitivity indices (TFQI, PTFQI, TT4RI, TSHI) are divided into four groups. According to the data in the table, we can see that the OR (95% CI) is increasing progressively (P<0.05), which indicates that the higher the thyroid hormone sensitivity impairment index, the higher the risk of hyperuricemia. **Table 2** shows that individuals in the highest group of thyroid hormone sensitivity index have a significantly higher incidence of hyperuricemia compared with those in the lowest group. [OR (95% CI): TFQI: 1.089 (1.059–1.119), P<0.001; PTFQI: 1.188 (1.020–1.539), P=0.013; TT4RI: 1.036 (1.020–1.052), P<0.001; TSHI: 1.050 (1.034–1.066), P<0.001].

**Table 2:** Odds ratios of sensitivity of thyroid hormone indices to risk of hyperuricemia by logistic regression analysis.

Item	Model 1		Model 2	
	OR (95% CI)	P value	OR (95% CI)	P value
TFQI				
Group 1 [-1, 0)	1 [Ref]		1 [Ref]	
Group 2 (0, 0.333]	1.034 (1.021–1.048)	<0.001	1.033 (1.020–1.046)	<0.001
Group 3 (0.333, 0.667]	1.055 (1.037–1.072)	<0.001	1.046 (1.030–1.062)	<0.001
Group 4 (0.667, 1.0]	1.092 (1.059–1.125)	<0.001	1.089 (1.059–1.119)	<0.001
PTFQI				
Group 1 [-1, 0)	1 [Ref]		1 [Ref]	
Group 2 (0, 0.333]	1.056 (1.038–1074)	<0.001	1.054 (1.038–1.071)	0.035
Group 3 (0.333, 0.667]	1.111 (1.022–1.207)	0.014	1.100 (1.018–1.187)	0.024
Group 4 (0.667, 1.0]	1.126 (1.021–1.492)	0.031	1.188 (1.020–1.539)	0.013
TT4RI				
Quartile 1 (-inf,1.478)	1 [Ref]		1 [Ref]	
Quartile 2 (1.478,2.147)	1.020 (1.005–1.039)	0.025	1. 016 (1.001–1.032)	0.029
Quartile 3 (2.147,2.816)	1.022(1.003–1.037)	0.022	1.020 (1.005–1.036)	0.010
Quartile 4 (2.816,inf)	1.022 (1.005–1.039)	0.011	1.036 (1.020–1.052)	<0.001
TSHI				
Quartile 1 (-inf,1.478)	1 [Ref]		1 [Ref]	
Quartile 2 (1.478,2.147)	1.030 (1.013–1.047)	<0.001	1.019 (1.004–1.035)	0.015
Quartile 3 (2.147,2.816)	1.048 (1.031–1.065)	<0.001	1.040 (1.024–1.056)	<0.001
Quartile 4 (2.816,inf)	1.051 (1.034–1.068)	<0.001	1.050 (1.034–1.066)	<0.001

Model 1, crude model; model 2, adjusted for sex; age (continuous); BMI, hypertension (categorical) and diabetes (categorical); OR, odds ratio; CI, confidence interval; TFQI, thyroid feedback quantile-based index; PTFQI, parametric thyroid feedback quantile-based index; TT4RI, thyrotrophic thyroxine resistance index; TSHI, thyroid-stimulating hormone index.

### Mediation analysis using serum total cholesterol

**Figure 3:**
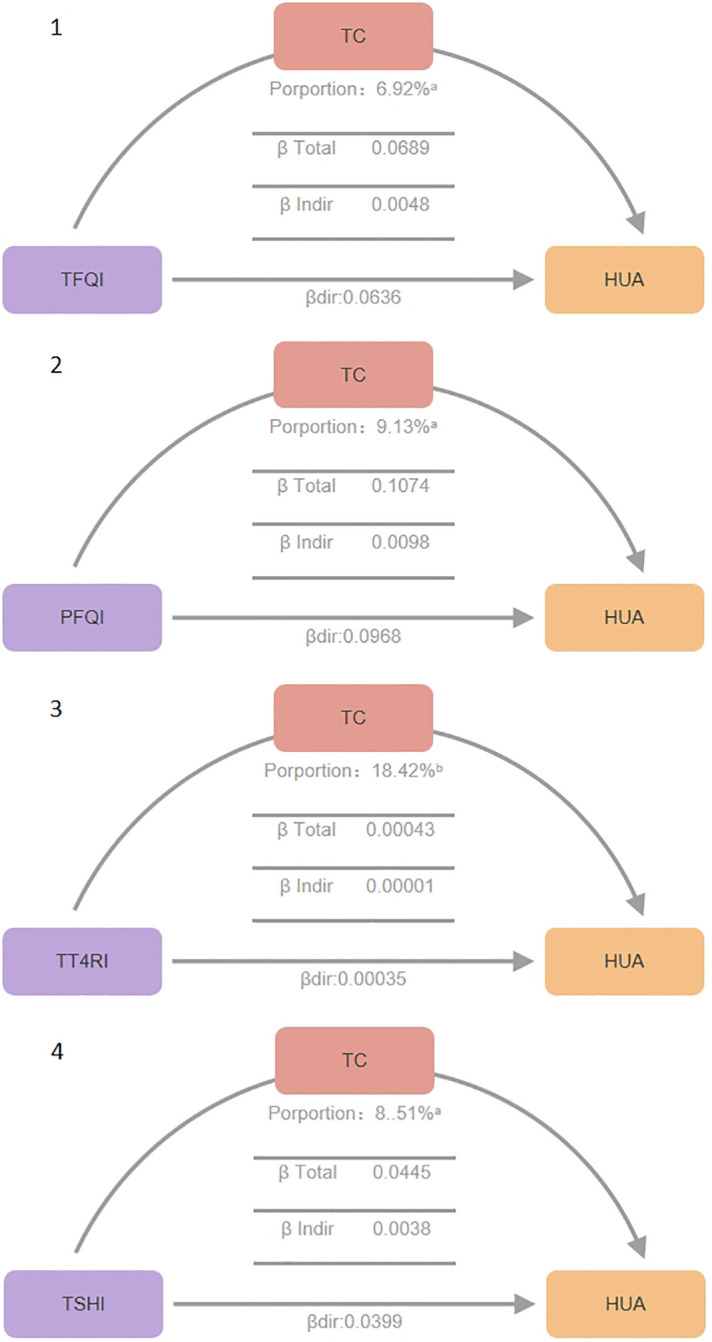
Mediation effect between thyroid hormone sensitivity impairment and hypercholesterolemia. Mediation analyses of the association between continuous thyroid hormones sensitivity indices and hyperuricemia through TC. HUA hyperuricemia, TC total cholesterol, TFQI thyroid feedback quantile-based index, PTFQI parametric thyroid feedback quantile-based index, TT4RI thyrotrophic thyroxine resistance index, TSHI thyroid-stimulating hormone index. a P value < 0.001, b P value < 0.05.

**Figure 3** summarizes the potential mediating effect of high total cholesterol between thyroid hormone sensitivity impairment and hyperuricemia.

Total Effect (β total), β total represents the overall impact of thyroid hormone sensitivity indices on hyperuricemia without considering mediating variables (total cholesterol). It is the sum of direct and indirect effects (β total = β dir + β indir). It reflects the combined direct and indirect effects of thyroid hormone sensitivity indices on uric acid levels. All four thyroid hormone sensitivity indices showed significant β total values (P<0.01), indicating plausible causal pathways. Specifically, TFQI β total: 0.0689 (P<0.001), PFQI β total: 0.1074 (P<0.001), TT4RI β total: 0.00043 (P=0.006), TSHI β total: 0.0445 (P<0.001).

Direct Effect (β dir),β dir represents the direct impact of thyroid hormone sensitivity indices on uric acid levels, after controlling for the mediating variable total cholesterol (TC).All four indices showed significant β dir values (P<0.05), indicating direct effects on serum uric acid levels even in the presence of mediating effects. TFQI β dir: 0.0048 (P<0.001), PFQI β dir: 0.0098 (P<0.001), TT4RI β dir: 0.00036 (P=0.026), TSHI β dir: 0.0038 (P<0.001).

Indirect Effect (β indir),β indir represents the indirect impact of thyroid hormone sensitivity indices on uric acid levels through the mediating variable total cholesterol. All four indices showed significant β indir values (P<0.01), confirming the mediating role of total cholesterol. TFQI β indir: 0.0636 (P<0.001), PFQI β indir: 0.0968 (P<0.001), TT4RI β indir: 0.00001 (P<0.001), TSHI β indir: 0.0400 (P<0.001).

Proportion Mediated, Proportion Mediated indicates the proportion of the total effect that is mediated through the mediating variable, reflecting the relative importance of the mediating pathway. TFQI Proportion: 6.92% (P<0.001), PFQI Proportion: 9.13% (P<0.001), TT4RI Proportion: 18.42% (P=0.006), TSHI Proportion: 8.51% (P<0.001).

Researchers conducted mediation analysis with BMI but did not find significant mediating effects. Mediation analysis with serum triglycerides also did not yield significant mediating effects.

## Discussion

This study investigated the revelation of serum total cholesterol (TC) as the mediator in the relationship between thyroid hormone sensitivity impairment and hyperuricemia (HUA) among a healthy population. Our findings introduce new epidemiological evidence for the multi-level interactive association of the thyroid-lipid metabolism-uric acid axis, indicating that thyroid hormone sensitivity status may indirectly affect uric acid homeostasis through the regulation of cholesterol metabolism. This breakthrough holds valuable implications for the early prevention of HUA and intervention strategies for subclinical thyroid dysfunction.

Thyroid hormones directly modulate key lipid metabolism genes (such as HMG-CoA reductase and LDL receptors) via nuclear receptors (TRs) (10). The positive correlation between thyroid hormone sensitivity indices and TC in this study suggests that thyroid resistance may induce elevated cholesterol synthesis and/or diminished clearance, which is consistent with the dyslipidemia patterns observed in patients with hypothyroidism (11). Notably, the mediating effect of TC (6.92-18.42%) reflects that cholesterol metabolism disorders may serve as an crucial bridge in the thyroid-uric acid axis. Potential mechanisms include: (1) Hypercholesterolemia may promote uric acid retention by upregulating renal urate reabsorption proteins (such as URAT1) (12, 13); (2) Oxidative stress and inflammation driven by abnormal TC may damage renal tubular function (14); (3) Cholesterol crystal deposition may directly cause renal interstitial injury (15).

This study expands upon previous research examining the relationship between thyroid dysfunction and HUA: 1. Contrary to the findings of Zhou Y et al. (16) in patients with thyroid disease, it can be observed that subclinical thyroid hormone sensitivity changes among a healthy population can influence uric acid metabolism via TC, highlighting the importance of early intervention. 2. Mediation analysis validated the “thyroid-lipid metabolism-uric acid” hypothesis proposed by Li J et al. (17), but identified TC, rather than LDL-C, as the primary mediator, possibly attributed to TC providing more comprehensively reflection of cholesterol metabolism status (18, 19). 3. Complementing animal studies (10, 20) where T3 directly inhibited XOD activity, this suggests that humans may possess a thyroid hormone sensitivity threshold effect, where direct uric acid regulation occurs only in the presence of significant dysfunction.

This study proposes: (1) Monitoring thyroid hormone sensitivity indices in high-risk HUA populations (such as those with metabolic syndrome) could facilitate early risk stratification; (2) Lipid-lowering treatment for patients with subclinical thyroid dysfunction may render “metabolic cascade benefits,” improving uric acid metabolism; (3) The applicability of current thyroid function reference ranges in assessing metabolic risk necessitates reevaluation (21).

It is important to note: (1) The cross-sectional design cannot establish causal relationships, and future cohort studies are necessary to verify the temporal relationship; (2) The exclusion of lipoprotein subfractions (such as oxidized LDL) and intestinal cholesterol absorption markers (such as plant sterols) may underestimate TC’s mediating effect; (3) Mendelian randomization studies are needed to exclude genetic confounding factors; (4) Animal experiments can further confirm the thyroid hormone receptor-specific regulatory pathways. Future research should focus on whether restoring thyroid hormone sensitivity (e.g., through lifestyle interventions) mitigates the risk of HUA by improving cholesterol metabolism, as well as the distinct impacts of different types of thyroid resistance (central *vs* peripheral).

Although this study adjusted for age, sex, BMI, diabetes, and hypertension, it did not account for important confounding factors such as dietary intake, alcohol use, renal function (eGFR), and physical activity. Owing to the absence of funding, the physical-examination dataset lacked records of these key variables; nevertheless, we intend to incorporate them into future longitudinal studies.

This study introduces a new association in a healthy population where thyroid hormone sensitivity impairment mediates the promotion of hyperuricemia through serum total cholesterol, thus, providing a theoretical foundation for the integrated prevention and treatment of metabolic diseases. Serum total cholesterol functions as a association mediator between thyroid hormone sensitivity impairment and hyperuricemia, with association involving lipid metabolism disorders, inflammation activation, and inhibition of renal uric acid excretion. Early lipid-lowering treatment for patients with thyroid dysfunction may produce “dual clinical benefits,” offering innovative perspectives for precision prevention and control of HUA. It is advisable to incorporate thyroid function sensitivity assessment into the management pathway for metabolic syndrome and explore multi-disease prevention strategies targeting cholesterol metabolism.

## Data Availability

The original contributions presented in the study are included in the article/supplementary material. Further inquiries can be directed to the corresponding author.
